# Retroviruses and reproduction revisited

**DOI:** 10.1007/s10815-018-1253-y

**Published:** 2018-07-13

**Authors:** Ann A. Kiessling

**Affiliations:** Bedford Research Foundation, 124 South Road, Bedford, MA 01730 USA

**Keywords:** Human immunodeficiency virus, Genetics, Acquired immunodeficiency disease, Semen, Condoms, Cytomegalovirus, Sexually transmitted disease, Assisted reproduction, Surrogacy

## Abstract

Thanks to effective anti-HIV medications, deaths from acquired immunodeficiency disease (AIDS) have plummeted, although the incidence of new HIV infections has decreased little, approximately 36,000 annually in the USA. The CDC estimates 1.1 million persons, mostly men, are living with HIV in the USA, with approximately 14% unaware they are infected. Since the global blood supply is essentially free of HIV today, infected semen is fueling the pandemic (88% of new infections in the USA), with needle sharing among IV drug abusers (7% of new US infections) and female to male transmission (5% of new infections) accounting for the balance. In spite of the importance to disease prevention and strategies for safe conception, semen transmission of HIV is not well understood. Because anti-HIV therapy does not eliminate HIV from semen, the Centers for Disease Control (CDC) for the past 25 years has espoused condom use as the safest approach to prevent HIV transmission, as well as other sexually transmitted diseases. A few months ago, however, an MMWR was circulated by the CDC that suggested condomless sex might be safe if the HIV-infected partner’s medications achieved an undetectable viral load in his blood. This new opinion was based on reports by three teams of investigators cited in the MMWR: “All three studies observed no HIV transmission to the uninfected partner while the partner with HIV was virologically suppressed with ART.” Unfortunately, this CDC statement does not fully describe the data presented in the studies, and abandoning condom use puts uninfected partners, including women seeking to conceive, at risk for infection by HIV and other STDs.

Nearly 30 years have passed since the initial discussion of the need to develop safe approaches to conception for women whose partners are infected with human immunodeficiency virus (HIV) [1]. In the interim, dozens of US fertility clinics have adopted guidelines for achieving safe conceptions with sperm from HIV-infected men [2, 3], whose numbers continue to grow in the USA.

Today, more than 75 million people have been HIV-infected worldwide, half of whom have died, and more than 73 HIV-vaccine trials have failed. Fortunately, thanks to the lobbying efforts of HIV-infected men and women on the NIH campus in the late 1980s, two dozen new anti-HIV medications were brought to clinical use in only two decades. This unprecedented rate of drug discovery was because the lobbying efforts reduced the review time for HIV grants from 9 to 6 months, and grant proposals could include actual drug discovery projects usually relegated to pharmaceutical companies.

Cocktails of anti-HIV medications referred to as “highly active antiretroviral therapy” (HAART) have dramatically reduced deaths from acquired immunodeficiency disease (AIDS), the inevitable result of untreated HIV infection. The incidence of new HIV infections has decreased little, however, approximately 36,000 annually in the USA. The CDC estimates 1.1 million persons, mostly men, are living with HIV in the USA, with approximately 14% unaware they are infected. Unlike the medications that actually cure hepatitis C virus infection, another virus for which there is no vaccine, HAART does not cure HIV infection; it inhibits new infections of immune cells by blocking various steps in the virus life cycle. Also unlike hepatitis C, HIV inserts a DNA copy, termed a provirus, of its RNA genetic information into the chromosomes of infected cells. Because cells infected by HIV may belong to the class of immune cells responsible for conferring life-long immunity, they remain in circulation in the body for decades. This is why HAART is considered to be a life-long necessity.

Since the global blood supply is essentially free of HIV today, infected semen is fueling the pandemic, accounting for 88% of new infections in the USA, approximately 90 per day. Needle sharing among IV drug abusers (7% of new infections in the USA) and female to male transmission (5% of new infections) account for the balance [4].

In spite of the importance to disease prevention, semen transmission of HIV is not well understood. HIV is present in semen as both virus particles containing HIV RNA, and infected cells containing HIV proviral DNA, and which form of the virus is principally responsible for semen transmission of infection is unknown. Moreover, since HIV can be detected in at least 50% of semen specimens from men naive to therapy, and in approximately 15% of specimens from men on HAART [5], it is difficult to understand the large epidemiologic studies that reveal sexual HIV transmission is relatively rare, on the order of 1/200 to 1/1000 sexual encounters [6]. Risk of transmission to an uninfected partner correlates with the level of measureable virus in the blood stream [7, 8]. For semen transmission to be so infrequent, it must depend either on semen virus titer, or the infected cells in semen, or specific circumstances in the recipient.

Several lines of evidence [9–12] indicate that semen-producing organs (testis, epididymis, vas deferens, seminal vesicles, prostate, urethra, and bulbourethral gland) comprise a separate compartment, “sanctuary sites,” of HIV infection that do not respond to HAART in parallel with the response in blood. Any one, or all, of these tissues could be a persistent focus of HIV infection. It takes only a few months for HAART to suppress HIV particles circulating in blood, the so-called viral load, but even after prolonged HAART, HIV and HIV-infected cells are occasionally ejaculated in semen [9–12]. Like the long-lived HIV-infected immune cells in blood, the long-lived infected immune cells in semen will be the most difficult to eradicate, since none of the current medications actually kill infected cells.

Moreover, other lines of evidence reveal that the subspecies of HIV in semen may be markedly different from the subspecies of virus in blood HIV [12], especially in men on HAART, and the virus particles in seminal plasma may not arise from the infected semen cells. The problem lies in the genetic complexity of HIV itself and the compartmentalization of virus between blood and semen. HIV’s strength and weakness are that on average, every virus particle has at least one mutation. Mutations are due in part to errors introduced by HIV’s reverse transcriptase when synthesizing the DNA provirus, but also by an innate cellular defense against HIV infection mediated by APOBEC3G deaminase [13]. These mutation pressures provide the framework for the spontaneous appearance of HIV quasispecies resistant to anti-viral medications, as well as the observed changeover in chemokine co-receptor specificity between the initial infecting virus (CCR5-tropic) and the resulting disease-promoting virus (CXCR4-tropic). This well-known switch in chemokine receptor specificity following infection of CD4+ cells [14] is due to mutations in the HIV gene that encodes its envelope protein. Additional information about the mechanism(s) of semen transmission is urgently needed for prevention of disease transmission [15].

Because anti-HIV therapy does not eliminate HIV from semen, the Centers for Disease Control (CDC) for the past 25 years has espoused condom use as the safest approach to prevent HIV transmission, as well as other sexually transmitted diseases, even by men with an undetectable burden of HIV in blood. More recently, uninfected partners of HIV-infected men have been prescribed “pre-exposure prophylaxis (PrEP),” an anti-viral regimen designed to prevent HIV transmission, although partners using PrEP are still counseled to use “safe sex” practices, and PrEP also does not block transmission of other sexually transmitted diseases, such as cytomegalovirus (CMV) and syphilis.

A few months ago, however, an MMWR was circulated by the CDC that suggested condomless sex might be safe if the HIV-infected partner’s HAART achieved an undetectable viral load in his blood [16]. This new opinion was based on reports by three teams of investigators cited in the MMWR: “All three studies observed no HIV transmission to the uninfected partner while the partner with HIV was virologically suppressed with ART [16].” Unfortunately, this statement by the CDC has spawned numerous “zero risk” for condomless sex website statements, such as:

“1. Can a person with HIV on treatment with an undetectable viral load transmit HIV?

No. A person living with HIV on antiretroviral therapy (ART) with an undetectable HIV viral load in their blood for at least six months cannot transmit HIV through sex. Sometimes the risk is described as “negligible” which means: so small as to not be worth considering; insignificant. Therefore, HIV experts and health educators have described the transmission risk in public health communications in clear and unambiguous ways such as: effectively no risk; untransmittable; no longer infectious; zero risk; no infection risk; do not transmit; cannot transmit.” [17]

In fact, contrary to the CDC statement, all three studies cited in the MMWR did report new HIV infections in the uninfected partner: 11 out of 1166 couples followed for 16 months [18], 78 out of 1763 heterosexual couples followed for 5 years [19], and 3 out of 358 homosexual couples followed for a year. But, by genetic analyses of blood virus in the newly infected partner, approaches questioned by other HIV experts [20], all of the new infections of partners were attributed by the authors of the three studies to sexual encounters outside the study couple.

Genetic analyses of HIV quasispecies in blood, which must be carefully and cautiously interpreted [21], have been helpful in tracking the spread of populations of HIV, but less helpful in tracking specific between-partner infections [22]. Because of the CCR5 tropism which persists in semen virus, even after the emergence of CXCR4-tropic virus in blood, and the highly discordant nature of virus subspecies between blood and semen, a more reliable way to trace between partner infection transmissions would be to compare the genetics of the new infection with semen virus from the infected partner. This was not the approach taken by the three groups of investigators referred to in the CDC’s MMWR [16].

An additional caution against relying on HAART to eliminate sexual transmission was recently reported in a UN-AIDS population study of an HIV endemic area in South Africa [23]. Following a concentrated effort to supply HAART to all HIV-infected persons in the study population, the prevalence of detectable viral load among HIV-infected persons fell from 73.7% in 2011 to 59.9% in 2014, but the hope for corresponding decrease in the prevalence of infection in the population was actually an increase, from 26.7% in 2011 to 32.3% in 2015.

Given this confusion, what counsel does the reproductive health care provider give patients? The most accurate information includes multiple points: (1) semen transmission of HIV is not well understood; (2) anti-viral therapy may not eliminate HIV from semen, but does statistically decrease the risk of transmission; (3) PrEP further reduces, but may not entirely eliminate, the risk of semen transmission; (4) sperm themselves are not HIV-infected, although HIV virus can “stick” to sperm [24]; (5) a meta-analysis of reported studies totaling 11,585 cycles of assisted reproduction with IUI or IVF for 3994 uninfected female partners of HIV-infected men revealed no reports of infection transmission [25]; (6) poor sperm quality in semen from HIV-infected men [26] increases the number of unprotected intercourses or inseminations necessary for conception; (7) until treatment strategies are developed to eliminate HIV from semen, condoms remain the safest prevention of HIV transmission, as well as blocking other sexually transmitted diseases.

Cytomegalovirus in semen is an increasing concern [27]. It is more common in semen of HIV-infected men and threefold more common than HIV. HAART does not effectively suppress CMV, which is one of the few viruses that can cross the placenta and infect the fetus. Importantly, it has not been possible to develop a fully protective vaccine against CMV, and having antibodies against CMV is not fully protective against a new infection during the first trimester of pregnancy that can be transmitted to the fetus [28, 29]. Syphilis is also on the rise. It is eightfold more prevalent in HIV-infected men than the general population [30].

All of these considerations suggest “condomless sex” with HIV-infected men will increase CMV and syphilis transmission, both serious considerations for women seeking pregnancy. Screening semen specimens, and the use of sperm from specimens with no detectable pathogen burden, either by timed insemination or by IVF [2], could thus maintain condom use and protect the woman from infection.

Surrogacy for HIV-infected men requires the highest bar for safety. Given the many unknowns surrounding HIV in semen and the inability to reliably test embryos for HIV infection [31], embryos created for transfer into a surrogate should be created with sperm from specimens with an undetectable burden of HIV [3]. Although the risk of an embryo infecting a surrogate is extremely low, until HAART strategies are developed that free semen entirely of HIV, surrogates deserve the most conservative safety measures possible and should not have to embark on an expensive, and perhaps uncomfortable, course of PrEP in addition to becoming pregnant. Although no reports of adverse effects of PrEP on fetal development have appeared, historically, some effects of medications given during pregnancy do not appear for a few decades (https://www.cancer.gov/about-cancer/causes-prevention/risk/hormones/des-fact-sheet). Moreover, concern about a possible CMV infection of a surrogate is highlighted by a 1992 court case of a surrogate undergoing an insemination becoming infected with CMV which was transmitted to the fetus (http://www.nytimes.com/1992/09/20/us/surrogate-mother-able-to-sue-for-negligence.html).

Full understanding of the mechanism(s) of semen transmission of infectious virus remains an urgent public health need—three decades after the onset of the HIV-pandemic. Counsel to couples seeking to conceive should include not only the process itself, but the continued use of condoms throughout the pregnancy to prevent infection by HIV, CMV, or other STDs.
